# Phytochemical Analysis, Antifungal, and Antioxidant Properties of Two Herbs (*Tristemma mauritianum* and *Crassocephalum bougheyanum*) and One Tree (*Lavigeria macrocarpa*) Species

**DOI:** 10.1155/2023/2565857

**Published:** 2023-01-25

**Authors:** Irene Chinda Kengne, Aimé Gabriel Fankam, Elodie Konack Yamako, Jean-De-Dieu Tamokou

**Affiliations:** ^1^Research Unit of Microbiology and Antimicrobial Substances, Faculty of Sciences, University of Dschang, Dschang, P.O. Box 67, Cameroon; ^2^Département des Sciences Appliquées à la Santé, Institut Universitaire et Stratégique de l'Estuaire (IUEs/Insam), BP 4100, Douala, Cameroon

## Abstract

Phytochemicals present in medicinal plants (herbs, shrubs, and trees) are endowed with high antimicrobial and antioxidant properties. The aim of this work was to study the chemical composition, antioxidant, and antifungal activities of *Tristemma mauritianum*, *Crassocephalum bougheyanum,* and *Lavigeria macrocarpa.* Chemical composition of the plant extracts was determined using standard methods. The antioxidant activities were performed using 2,2-diphenyl-1-picrylhydrazyl (DPPH), ferric reducing antioxidant power (FRAP), nitric oxide (NO), and hydroxyl (OH) scavenging assays. The antifungal activity of plant extracts and their combinations with antifungals was evaluated against eleven *Candida spp*. using the broth microdilution method by determining the minimum inhibitory concentration (MIC) and minimum fungicidal concentration (MFC). The quantitative chemical analysis of the extracts of *T. mauritianum*, *L. macrocarpa,* and *C. bougheyanum* showed that they contain phenols, tannins, and flavonoids that vary according to the plant species and extracts. All the plant extracts presented promising antifungal (MIC = 64–2048 *µ*g/mL) and antioxidant activities. The extract of *T. mauritianum* displayed the highest antifungal (MIC = 64–256 *µ*g/mL) and antioxidant (IC_50_ = 19.052 ± 1.11 *μ*g/mL) activities which can be explained by its high phenolic content. Interestingly, extracts of *T. mauritianum*, *L. macrocarpa*, and *C. bougheyanum* displayed synergistic effects (fractional inhibitory concentration index, FICI ≤ 0.5) with ketoconazole against clinical resistant isolates. The results of the present study demonstrate promising antifungal and antioxidant activities of the tested plants that are associated to their phenol, tannin, and flavonoid contents. Hence, extracts of *T. mauritianum* and *L. macrocarpa* could be deeply investigated as antifungal alone and in combination with conventional antifungal drugs to treat infections caused by *Candida spp*.

## 1. Introduction

Infectious diseases appear as a major cause of mortality and morbidity in humans. Amongst them, invasive fungal infections have dramatically increased mainly in immunocompromised individuals [1]. *Candida* species that are the most common isolated in clinical fungal invasive infection are *C. albicans*, *C. tropicalis*, *C. parapsilosis*, and *C. glabrata* [2]. Despite current antifungal therapies, *Candida* infections show unpleasant high mortality rates. For example, the previous study indicated that mortality attributable to invasive *Candida* infections was about 19–24% among hospitalized patients [3]. Limited antifungal arsenal, diverse side effects, and drug-resistant strains appear as the main factors that contribute to that scenario [4]. In recent years, the emergence of clinically resistant strains is a major cause of failure in the treatment of invasive fungal infections. The use of prolonged or repeated treatment with antifungals, such as fluconazole, is responsible for this scenario [5]. Therefore, the search for new antifungals is necessary and natural sources deserve attention because of the perception that they cause minimal side effects and have a long history of use in folk medicine for the treatment of fungal infections and oxidative stress conditions [6, 7]. Low levels of reactive oxygen species (ROS) or free radicals are essential for cells to carry out normal biochemical functions such as cell signalling and apoptosis of defective cells [8]. Therefore, excessive generation of free radicals damages biological components that lead to aging and several chronic diseases such as cancer and cardiovascular diseases in humans [9]. Although an endogenous system of antioxidant is present in our body to get rid of excessive free radicals, exogenous antioxidants are recommended [10]. Chemically synthesized antioxidant compounds such as butylated hydroxytoluene have been questioned due to reports of their carcinogenicity [11]. Therefore, alternative antioxidants that have minimal side effects are highly needed. The bioactive constituents extracted from the root and above-ground biomass of medicinal plants contain secondary metabolites (also known as phytochemicals), which represent a diverse group of natural products including alkaloids, phenols, flavonoids, terpenoids, steroids, saponins, tannins, quinones, coumarins, and glycosides [12]. Phenolic compounds (including flavonoids and tannins) are the most abundant phytochemicals in plant kingdom that serve as a supply source of health-beneficial properties such as antimicrobial and antioxidant activities in the human diet [12, 13].

*Crassocephalum bougheyanum* (Compositae) is a flowering herb from subtropical or tropical dry forest and is commonly found in Cameroon [14]. In Cameroon, Congo, Gabon, and Nigeria, it is used as medicines and vegetables [15]. Essential oils obtained from *C. bougheyanum* had shown to contain *α*-phellandrene, p-cymene, pinenes, myrcene, limonene, and (E)-*β*-ocimene, which are all monoterpene hydrocarbons [16]. *Lavigeria macrocarpa* (Icacinaceae) is a shrub having an extensive liana of 24 m long cauliflorous, with a large underground tuber and rusty stellate pubescent and ecliptic leaves [17]. *L. macrocarpa* is mostly found in Cameroon forest area. It is used to treat rheumatism, poisoning, snakebites, malaria fever, and other feverish conditions [18, 19]. *Tristemma mauritianum* (Melastomataceae) is a plant with a height of 1.25 m, and it is mostly found in marshy and moist places such as Senegal, West Cameroon, Equatorial Guinea, Congo Brazzaville, and upper Shari [20]. *T. mauritianum* is also used to treat oral and digestive candidiasis in children, paralysis, epilepsy, convulsions, and spasm [21]. Stem and leaf decoction of *T. mauritianum* is used to treat diarrhea, dysentery, and skin infections [22, 23]. Previous research has shown its antisalmonella and antioxidant properties [24]. The GS/MS analysis has shown that *T. mauritianum* contains 2,4-di-*tert*-butylphenol, 2-((octyloxy) carbonyl) benzoic acid, and sitosterol [25]. The aim of this work was to study the chemical composition, antioxidant, and anticandidal activities of *T. mauritianum* J. F. (Gmel), *C. bougheyanum* C. D. Adams, and *L. macrocarpa* (Oliv.) Pierre.

## 2. Materials and Methods

### 2.1. Plant Materials

The plants used in this study were the aerial parts of *Tristemma mauritianum* J.F. (Gmel) (Mecastomataceae); *Crassocephalum bougheyanum* C.D. Adams (Compositae), and the leaves and roots of *Larvigeria macrocarpa* (Oliv.) Pierre (Icacinaceae). These plants were harvested on October 2019 in Tombel subdivision, Kupe Muanenguba division, South-West region, Cameroon (4°44′47″N/9°40′13″E). The plant species were identified and authenticated at the Cameroon National Herbarium, where the voucher specimens were kept under the references 6995/SRF-Cam, 7635/HNC-Cam, and 179761/SRF for *T. mauritianum*, *C. bougheyanum*, and *L. macrocarpa,* respectively.

### 2.2. Preparation of Extracts

The plant materials were washed thoroughly under running water, air-dried under room temperature, and crushed to powder using mixer-grinder. The air-dried and powdered material from each plant was soaked separately in methanol (1/4 w/v) for 48 h at room temperature with shaking five times per day. The mixture was filtered through a Whatman filter paper No. 1, and the filtrate was concentrated by evaporation at 65°C using a rotatory evaporator (Buchi R-200) to obtain the crude extract that was dried in an oven at 40°C. The extract was finally kept at +4°C until further use.

### 2.3. Phytochemical Analysis of the Plant Extracts

#### 2.3.1. Qualitative Phytochemical Screening

Standard methods described by Harbone [26] were used to perform the qualitative phytochemical screening of plant extracts. The various plant extracts were screened for the presence of triterpenes, steroids, phenols, saponins, tannins, flavonoids, anthraquinones, and alkaloids.

#### 2.3.2. Determination of Total Phenolic Content (TPC)

The total phenolic content (TPC) was determined as described by Ramde-Tiendrebeogo et al. [27]. The reaction mixture in this test consisted of 20 *µ*L of extracts (2 mg/mL), 100 *µ*L of the Folin-Ciocalteu reagent (diluted 10 times in water), and 80 *µ*L of a sodium carbonate solution 20%. The mixture was stirred and incubated in a water bath at 20°C for 30 min, and then the absorbance was measured with a spectrophotometer (Biobase Bk-D590 Double Beam Scanning UV/Vis) at 765 nm. The extracts were replaced with distilled water for control tubes. A calibration curve was plotted using gallic acid (concentrations ranged from 0.015 to 2 mg/mL). Results were expressed as milligram of gallic acid equivalent per gram of extract (mg·GAE/g).

#### 2.3.3. Determination of the Total Flavonoid Content (TFC)

The total flavonoid content (TFC) of the extracts was determined using the aluminium chloride colorimetric method [28]. A volume of 100 *µ*L of extracts (2 mg/mL) was mixed with 50 *µ*L of aluminium chloride (1.2%), and then 50 *µ*L of potassium acetate (120 mM) was added. The mixture was incubated for 30 min at room temperature, and the absorbance was measured with a spectrophotometer (Biobase Bk-D590 Double Beam Scanning UV/Vis) at 415 nm. The extracts were replaced with distilled water for control tubes. TFC was calculated using the quercetin calibration curve (concentrations ranged from 0.015 to 2 mg/mL), and results were expressed as milligram quercetin equivalent per gram of extract (mg·QE/g).

#### 2.3.4. Determination of the Total Tannin Content (TTC)

The total tannin content (TTC) of the extract was determined using the Folin–Ciocalteu method as previously described [29]. The reaction mixture consisted of 100 *µ*L of extracts (2 mg/mL), 500 *µ*L of the Folin–Ciocalteu reagent (diluted 10 times in distilled water), 1000 *µ*L of sodium carbonate solution at 35%, and 8.4 mL of distilled water. The mixture was stirred and incubated at room temperature for 30 minutes, and then the absorbance was measured in a spectrophotometer (Biobase Bk-D590 Double Beam Scanning UV/Vis) at 700 nm. The extracts were replaced with distilled water for control tubes. A calibration curve was plotted using tannic acid (concentrations ranged from 100 to 500 *µ*g/mL). The results were expressed in milligram equivalent of tannic acid per gram of extract (mg·TAE/g).

### 2.4. Antioxidant Assays

#### 2.4.1. DPPH Radical Scavenging Assay

The antiradical activity of each plant extract was evaluated using the protocol described previously [30]. Briefly, a volume of 900 *μ*L of DPPH methanol solution (20 mg/L) was mixed with 100 *μ*L of test sample. The samples were prepared in methanol and tested at concentration range of 12.5 to 200 *μ*g/mL. The mixture was incubated in a dark room at room temperature for 30 minutes and the absorbance was read in spectrophotometer (Biobase Bk-D590 Double Beam Scanning UV/Vis) at 517 nm. L-ascorbic acid was used as a standard antioxidant (12.5 to 200 *µ*g/mL). The experiments were carried out in triplicate for each concentration. The optical densities obtained were converted to percentage inhibition, and the percentages of DPPH° scavenged (%RSa) by test samples were calculated as %RSa = [(*A*_0_ − *A*_1_)/*A*_0_] × 100, where *A*_0_ is the absorbance of the DPPH alone and *A*_0_ is the absorbance of the mixture. The half-maximal inhibitory concentration (IC_50_) values were estimated from the %RSa versus log of concentration plots using a nonlinear regression algorithm.

#### 2.4.2. Ferric Reducing Assay

The reducing power of plant extracts was determined by applying the method described previously [31]. Briefly, 1 mL of each plant extract at different concentrations (200, 100, 50, 25, and 12.5 *µ*g/mL) was mixed with 2.5 mL of a 0.2 M phosphate buffer solution (pH 6.6) and 2.5 mL of 1% potassium ferricyanide (K_3_Fe(CN)_6_). The resulting solution was incubated in a water bath at 50°C for 20 min. Then, 2.5 mL of 10% trichloroacetic acid was added to stop the reaction, and the tubes were centrifuged at 300 rpm for 10 min. An aliquot (2.5 mL) of the supernatant was mixed with 2.5 mL distilled water and 0.5 mL of 0.1% FeCl_3_ methanol solution. The absorbance was read at 700 nm using a spectrophotometer (Biobase Bk-D590 Double Beam Scanning UV/Vis). Vitamin C was used as a standard antioxidant (12.5 to 200 *µ*g/mL).

#### 2.4.3. Hydroxyl Radical Scavenging Assay

The hydroxyl scavenging activity of the extracts was determined using Fenton reaction as previously described [32]. Briefly, 60 *µ*L of FeCl_3_ was mixed with 90 *µ*L of 1,10-phenanthrolin (1 mM). Then, 2.4 mL of phosphate buffer (0.2 M, pH 7.4) and 150 *µ*L of H_2_O_2_ (0.17 M) was added. A volume of 1.5 mL of extract at concentrations ranging from 12.5 to 200 *µ*g/mL was introduced in the mixture. The reacting mixture was incubated for 5 min at room temperature. After incubation, the absorbance was read at 560 nm in a spectrophotometer (Biobase Bk-D590 Double Beam Scanning UV/Vis). Vitamin C was used a as standard antioxidant (12.5 to 200 *µ*g/mL). The percentage of hydroxyl radical scavenging activity (%HRSA) is calculated by the following formula: %HRSA = [(*A*_0_ − *A*_1_)/*A*_0_] × 100, where *A*_0_ is the absorbance of the control and *A*_1_ is the absorbance of the mixture.

#### 2.4.4. Nitric Oxide Scavenging Assay

Nitric oxide scavenging activity of the extracts was carried out as previously described [33] with some modifications. In a quartz cuvette, to 0.75 mL of sodium nitroprusside(10 mM) in phosphate buffer, 0.5 mL of extract/standard (vitamin C) (concentrations varying from 12.5 to 200 *µ*g/mL) was added. The resulting mixture was incubated at room temperature for 60 min. Then, 1.2 mL of Griess reagent (10% sulfanilamide in 5% phosphoric acid and 0.1% N-(1-napthyl) ethylenediamine dihydrochloride in distilled water) was added. The final concentration varied between 12.5 and 200 *µ*g/mL. After 5 min of incubation in a dark room at room temperature, the absorbance of chromophore formed was read using a spectrophotometer (Biobase Bk-D590 Double Beam Scanning UV/Vis) at 540 nm. The control tubes contained methanol rather than extracts. The radical scavenging percentage (NRS) of the various extract was calculated as follows: NRS (%) = [(*A*_0_ − *A*_1_)/*A*_0_] × 100, where *A*_0_ is the absorbance of the control and *A*_1_ is the absorbance of the extract/standard.

### 2.5. Antifungal Assay

#### 2.5.1. Microorganisms and Culture Conditions

Eleven (11) *Candida* strains/clinical isolates were used for this study. They included *C. dubliniensis* (*C. dubliniensis* 5 : I52*, C. dubliniensis* 1 : I59, and *C. dubliniensis* 3 : I81)*, C. albicans* (*C. albicans* ATCC10231, *C. albicans Cppc* BACT 017*, C. albicans* 7Ca*, C. albicans* 11Ca, and *C. albicans* 18Ca)*, C. glabrata* (*C. glabrata* 11 : I81 and *C. glabrata* 10 : I39), and *C. tropicalis Cpc* BACT 018 (Table S1). These microorganisms were collected from the Research Unit of Microbiology and Antimicrobial Substances (Cameroon). They were maintained on sabouraud dextrose agar (SDA) at 4°C and subcultured on a fresh SDA for 48 h prior to any antifungal assay.

#### 2.5.2. Determination of Minimum Inhibitory Concentration (MIC) and Minimum Fungicidal Concentration (MFC)

The minimal inhibitory concentrations (MICs) of extracts against the yeast species were determined by the broth microdilution method recommended by the Clinical and Laboratory Standards Institute [34] with slight modifications. Briefly, extracts were first dissolved in dimethylsulfoxide/sabouraud dextrose broth (DMSO/SDB, 1 : 1 V/V) and serially diluted two-fold in SDB, making each well to 100 *μ*L (in a 96-wells microplate). One hundred microliters (100 *μ*L) of inoculum (1.5 × 10^6^ CFU/mL) prepared in SDB were then added. Wells containing SDB, inoculum, and DMSO at a final concentration of 1% served as negative control. Fluconazole and ketoconazole (Merck, Darmstadt, Germany) were used as reference antifungal drugs (concentrations varying from 0.0313 to 512 *µ*g/mL). Each plate was covered with a sterile plate sealer, gently shaken to mix the contents of the wells followed by incubation at 35°C. The MICs were then assessed visually and were taken as the lowest sample concentration inhibiting the growth of the microorganism. The minimum fungicidal concentrations (MFCs) were determined by adding 50 *µ*L aliquots of the preparations, which did not show any yeast growth after incubation during MIC assays, into 150 *µ*L of SDB. These preparations were further incubated at 35°C for 48 hrs. The lowest concentration that showed no visual growth after the subculturing was considered as the minimum fungicidal concentration (MFC). The experiment was performed in triplicate and repeated three times for both MIC and MFC determinations with similar results.

#### 2.5.3. Evaluation of the Combination Effects of Ketoconazole/Fluconazole with Extracts

The effects of a combination of antibiotics (ketoconazole and fluconazole) with extracts of *T. mauritianum*, *L. macrocarpa,* and *C. bougheyanum* against 10 pathogenic yeasts were assessed by the Checkerboard method as previously described [35]. The drug-resistant yeasts were inoculated into a 96-well microtitre plates, and a serial dilution of antibiotics and extracts was performed. Each well consisted of unique combination of test extract and antifungal drug. The plates were then incubated for 48 h at 35°C. The analyses were performed in triplicates. The interactions between the antifungal agents were evaluated by calculating the fractional inhibitory concentration indices (ΣFIC) at their subinhibitory concentrations. The ΣFIC is defined as follows: MIC of antifungal tested in combination/MIC of antifungal tested alone + MIC of extract tested in combination/MIC of extract tested alone. The FIC index is interpreted as ΣFIC ≤ 0.5: synergistic effect, 0.5 < ΣFIC ≤ 1: additive effect, 1 < ΣFIC ≤ 2: indifferent effect, and ΣFIC > 2.0: antagonistic effect.

### 2.6. Statistical Analysis

The data obtained were analysed using one-way analysis of variance (ANOVA) and presented as mean ± standard deviation (SD) of three replications. The levels of significance, considered at *p* < 0.05, were determined by Waller–Duncan test using the Statistical Package for the Social Sciences (SPSS) software version 22.0.

## 3. Results

### 3.1. Qualitative Phytochemical Analysis

The presence of major phytochemical groups was detected in the studied plant extracts. The results indicated that all the extracts contain steroids, flavonoids, phenols, and tannins (Table 1). The leaves and roots of *L. macrocarpa* contain all the class of phytochemicals except anthraquinones, which are present only in *T. mauritianum*. All the classes of phytochemicals except triterpenes were present in the extract of *C. bougheyanum* (Table 1).

### 3.2. Quantitative Phytochemical Analysis of Extracts

The total phenolic, flavonoid, and tannin contents of the studied plant extracts were evaluated. The results obtained show a variation in total phenolic, flavonoid, and tannin contents depending on the plant extracts (Table 2). The extract of *T. mauritianum* presented the highest phenolic content (110.23 ± 0.13 mg·GAE/g), followed in decreasing order by those of *C. bougheyanum* (61.82 ± 0.14 mg·GAE/g), *L. macrocarpa* leaves (57.89 ± 0.48 mg·GAE/g), and *L. macrocarpa* roots (41.30 ± 0.39 mg·GAE/g).

The extract of *L*. *macrocarpa* leaves presented the highest flavonoid content (9.16 ± 0.10 mg·CE/g), followed in decreasing order by those of *T. mauritianum* (7.32 ± 0.06 mg·CE/g), *L*. *macrocarpa* roots (7.11 ± 0.10 mg·CE/g), and *C. bougheyanum* (6.57 ± 0.14 mg·CE/g). The extract of *T. mauritianum* also recorded the highest tannin content (10.90 ± 0.52 mg·TAE/g), followed in decreasing order by those of *C. bougheyanum* (9.08 ± 0.62 mg·TAE/g), *L. macrocarpa* roots (6.14 ± 0.13 mg·TAE/g), and *L. macrocarpa* leaves (4.08 ± 0.18 mg·TAE/g).

### 3.3. Antioxidant Activity

#### 3.3.1. DPPH Radical Scavenging Activity

**Figure 1:**
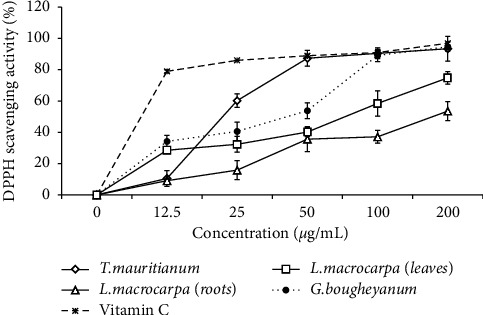
DPPH radical scavenging activity of plant extracts and vitamin C as a function of concentrations.

The capacity of the extract to scavenge the DPPH radical was determined and expressed in IC_50_. It was observed that all the extracts possess a concentration-dependent antiradical activity (Figure 1 and Table 3). Apart from the root extract of *L. macrocarpa,* all the extracts inhibit significantly (*p* < 0.05) the DPPH radical. The extract of *C. bougheyanum* presented the highest percentage of DPPH radical scavenging activity mainly at a concentration of 200 *µ*g/mL (94.69 ± 2.98%) followed in decreasing order by those of *T. mauritianum* (93.42 ± 7.85%) and *L. macrocarpa* leaves (74.83 ± 4.05%) (Figure 1). Based on the calculated IC_50_ values, extracts showed medium-to-strong DPPH scavenging activity ranging from 19.05 to 259.21 *μ*g/mL. The extract of *T. mauritianum* showed the most potent antioxidant activity (IC_50_ = 19.052 ± 1.11 *μ*g/mL) followed by that of *C. bougheyanum* (IC_50_ = 23.70 ± 1.18 *μ*g/mL). Vitamin C used as standard antioxidant was globally more potent than the extracts at all the tested concentrations, with IC_50_ of 8.13 ± 1.78 *μ*g/mL (Table 3).

#### 3.3.2. Ferric Reducing Antioxidant Power (FRAP) of Extracts

**Figure 2:**
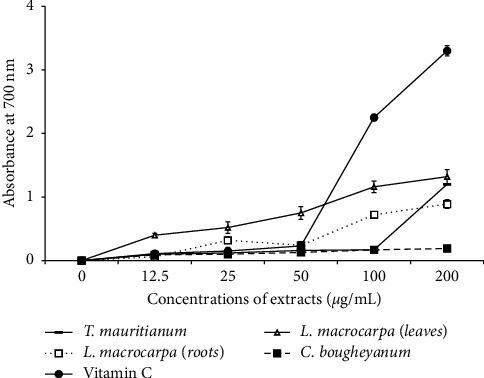
Ferric reduction ability of plant extracts as a function of concentrations.

The reducing power of iron was determined by the transformation of Fe^3+^ into Fe^2+^ in the presence of extracts and expressed as any increase in absorbance at 700 nm. The results showed that the extract of *L. macrocarpa* leaves presented the best reducing power at all tested concentration compared to other extracts. Vitamin C used as standard drug presented the highest ferric reducing activity than that of the plant extracts at concentration of 200 *µ*g/mL (Figure 2).

#### 3.3.3. Hydroxyl Radical Scavenging Activity

The hydroxyl radical scavenging activities of the selected plant extracts are presented in Table 4. It was observed that the extract of *C. bougheyanum* showed the highest inhibition percentage (94.03%) at a concentration of 200 *µ*g/mL than that of other extracts, not significantly different (*p* ≥ 0.05) from that of the reference antioxidant, vitamin C which presented percentage inhibition of 96.98% at the same concentration.

#### 3.3.4. Nitric Oxide (NO) Scavenging Activity

The extracts were evaluated for their ability to scavenge the nitric oxide (NO) radical. The results have shown that all the extracts are able to inhibit the NO radical with inhibitory potential that varies from one extract to another (Table 5). Apart from the extract of *T. mauritianum*, all the extracts inhibited the NO radical by more than 50% at all the tested concentrations. Moreover, *L. macrocarpa* leaf extract presented the highest nitric oxide scavenging capacity at all the concentrations compared to the other extracts (78.42 to 96.09%). Vitamin C displayed the highest activity at all the tested concentrations.

### 3.4. Antifungal Activity

#### 3.4.1. Minimum Inhibitory Concentration and Minimum Fungicidal Concentration

The MIC and MFC values were determined to evaluate the antibacterial activities of the studied plant extracts. The results presented in Table 6 show that plant extracts exhibit variable antifungal activities against the tested yeasts, with MIC values ranging from 64 *µ*g/mL to 2048 *µ*g/mL for extracts and 1 *µ*g/mL to 256 *µ*g/mL for antifungal drugs. The extract of *T. mauritianum* was the most active with the lowest MIC value of 64 *µ*g/mL against *C. dubliniensis* 1:I59, *C. albicans* ATCC 10231, *C. albicans* 18Ca, and *C. tropicalis* Cpc BACT 018. It was 100% (11/11) active against the tested yeasts followed in decreasing order by those of *L. macrocarpa* (leaves), *G. bougheyanum*, and *L. macrocarpa* (roots). MFC values varied from 256 *µ*g/mL to 2048 *µ*g/mL for the tested extracts and 1 *µ*g/mL to 256 *µ*g/mL for antifungal drugs.

#### 3.4.2. Combination Effects of Ketoconazole/Fluconazole with the Plant Extracts

The results of the interaction study between known antifungal drugs (ketoconazole and fluconazole) and the tested plant extracts at their subinhibitory concentrations against yeast species are presented in Tables 7 and 8. Globally, we found that all the extracts showed synergistic effect with ketoconazole/fluconazole against at least two yeast species. Synergy (∑FIC ≤ 0.5) was observed for the combinations of ketoconazole with the extracts of *L. macrocarpa, T. mauritianum* (leaves), and *C. bougheyanum* against five (50%), four (40%), and three (30%) of the ten drug-resistant yeasts tested, respectively. Moreover, *C. bougheyanum* displayed 4 cases (40%) of additive effects (0.5 < ΣFIC ≤ 1) in combination with ketoconazole (Table 7). Interactions between the tested extracts and fluconazole were mainly indifferent effects (1 < ΣFIC ≤ 2) whereas some synergistic and additive interactions were noted for these combinations. For example, synergistic effects were observed against 3 (30%) and 2 (20%) out of 10 drug-resistant yeasts tested, with the combinations of fluconazole with the extracts of *L. macrocarpa, T. mauritianum* (leaves), and *C. bougheyanum*, respectively (Table 8). No antagonistic effect was recorded with the combination of antifungal drugs and plant extracts.

## 4. Discussion

Phytomedicines have become increasingly popular for their potential use in curing many kinds of ailments with higher therapeutic value, lower toxicity, and fewer side effects when compared to allopathic medicines [36]. In the current study, the quantitative chemical analysis of the plant extracts was carried out with the aim of determining the content of secondary metabolites, which could explain their antioxidant and antifungal activities. In fact, the biological activity of medicinal plants is correlated with the presence and level of one or more classes of bioactive secondary metabolites [37, 38]. The results of this work indicate that all the studied plant extracts contain steroids and phenols such as flavonoids and tannins. In comparison to our results, bioguided fractionation of the MeOH extract of *T. mauritianum* aerial parts led to the identification of luteolin-3′-O-*β*-D-glucuronopyranosyl butyl ester, quercetin-3-O-*β*-D-glucuronopyranosyl butyl ester, arjunolic acid-28-*β*-D-glucopyranosyl ester (Arjunglucoside II), asiatic acid-28-*β*-D-glucopyranosyl ester (Quadranoside IV), *β*-sitosterol, oleanolic acid, ellagic acid, casuarinine, luteolin, pterocaryanin C, quercetin-3-O-*β*-D-glucopyranoside, and 6-hydroxyapigenin-7-O-*β*-D-glucopyranoside [39]. Other study has found that *T. mauritianum* extract possesses significant quantities of phenols and flavonoids [25]. However, this is a pioneer study performing the phytochemical composition of *C. bougheyanum* and *L. macrocarpa* extracts.

As multiple mechanisms are involved in the initiation of the oxidative stress, a single method is not sufficient to conclude about the antioxidant property of a sample [40]. Hence, in this work, the antioxidant activity of the plant extracts was confirmed by four tests (DPPH, NO, OH, and FRAP) even at low concentrations. Indeed, the DPPH radical scavenging assay is based on the ability of the stable free radical 2,2-diphenyl-1picrylhydrazyl to react with hydrogen donors including phenolic acids, flavonoids, and tannins [41, 42]. In the physiology condition, the interaction between ferric ion and superoxide anion induced the formation of hydroxyl radical, which induced the oxidation of DNA, lipid peroxidation, oxidation of proteins, and the activation of kinases [43]. Thus, the reduction of ferric ion can prevent those damages. The presence of reductants (antioxidants) in the tested plant extracts can cause the reduction of Fe^3+^/ferrocyanide complex to ferrous form [44]. Hydroxyl radical is the major active oxygen species that causes lipid oxidation and important biological damage reacting with polypeptides, saccharides, nucleotides, and organic acids [45]. The role of the free radical (NO) in inflammatory processes is well known [46]. Total phenols, flavonoids, and tannins can be linked to the antioxidant properties of the tested plants by acting as reducing agents, hydrogen donors, and singlet oxygen quenchers. Other groups of compounds that possess antioxidant activity, such as alkaloids, can contribute to this antioxidant potency. Indeed, it is well documented that synergies between various chemicals must be taken into consideration when predicting their biological activities [47, 48]. An extract is considered as having significant antioxidant potential when IC_50_ < 20 *µ*g/mL, moderate when 20 ≤ IC_50_ ≤ 75 *µ*g/mL, and weak when IC_50_ > 75 *µ*g/mL [49]. Based on that, extracts of *T. mauritianum* (IC_50_ = 19.052 ± 1.11 *μ*g/mL) and *C. bougheyanum* (IC_50_ = 23.70 ± 1.18 *μ*g/mL) have significant antioxidant activity. These results are highly supported by the quantities of TPC and TFC present in these extracts.

The findings of this study showed that *T. mauritianum, C. bougheyanum*, and *L. macrocarpa* exhibited variable antifungal activities against the tested microorganisms, with MIC values ranging from 64 *µ*g/mL to 2048 *µ*g/mL which can be linked to the plant species, part of the plant analysed, and the presence of secondary metabolites. In fact, phenols, flavonoids, and tannins have been found to be active on pathogenic microorganisms [50–52]. According to Tamokou et al. [53], the activity of plant extracts is classified as significant (MIC < 100 *µ*g/ml), moderate (100 < MIC ≤ 625 *µ*g/ml), or weak (MIC > 625 *µ*g/ml). Hence, the extract of *T. mauritianum* was significantly active, with the MIC value of 64 *µ*g/mL against *C. dubliniensis* 1:I59, *C. albicans* ATCC 10231, *C. albicans* 18Ca, and *C. tropicalis* Cpc BACT 018.

The extracts of *L. macrocarpa* (leaves) and *C. bougheyanum* (aerial parts) displayed no MFC values against all the tested yeast species on which MIC values were determined, indicating that these extracts have fungistatic effect (MFC/MIC > 4) [7]. However, the extract of *T. mauritianum* (aerial parts) showed fungicidal activity (MFC/MIC ≤ 4) against *C. dubliniensis* 5 : 152 and *C. albicans* ATCC 10231 while that of the roots of *L. macrocarpa* showed fungicidal effect (MFC/MIC ≤ 4) against *C. albicans* ATCC 10231 and *C. albicans* 7Ca.

With the increased incidence of drug-resistant fungi, synergistic combinations have been explored between conventional drugs and natural bioactive substances resulting in a new direction in antifungal drug discovery and antifungal therapy. Hence, research on the use of combinations of antifungals with natural substances to overcome fungal resistance has attracted considerable attention [1, 2]. In this study, the extract of *L. macrocarpa* displayed the most relevant synergistic effect (fractional inhibitory concentration index, FICI ≤ 0.5) with ketoconazole against 50% clinical resistant isolates. This result suggests that the combinations of the extract of *L. macrocarpa* with antifungal drugs could be an alternative to treat invasive fungal infections involving drug-resistant*Candida spp*. The current findings also indicated that the tested extracts and mainly that of *T. mauritianum* have promising antifungal activity, which might be attributed to the presence of phenols, especially flavonoid and tannin contents. Overall, the results of the current study are in agreement with those of Ngoudjou et al. [25] who demonstrated that *T. mauritianum* extracts had significant antioxidant and antibacterial activities. To our knowledge, this is the first study showing anticandidal activity of *T. mauritianum.* Additionally, this is a pioneer study demonstrating the antioxidant and antifungal activities of *L. macrocarpa* and *C. bougheyanum* and the synergistic effect between these plant species and ketoconazole.

## 5. Conclusion

The results of the present study demonstrate the antifungal and antioxidant activities of the tested plants that could be attributed to their phenolic contents. Hence, extracts of *T. mauritianum* and *L. macrocarpa* could be deeply investigated as antifungal alone and in combination with conventional antifungal drugs to treat infections caused by *Candida spp*. [54].

## Figures and Tables

**Table 1:** Major phytochemical groups of the studied plant extracts.

Phytochemical groups	*T. mauritianum* (aerial parts)	*L. macrocarpa* (leaves)	*L. macrocarpa* (roots)	*C. bougheyanum* (aerial parts)
Flavonoids	+	+	+	+
Phenols	+	+	+	+
Tannins	+	+	+	+
Alkaloids	−	+	+	+
Saponins	+	+	+	+
Anthraquinones	+	−	−	−
Triterpenes	−	+	+	−
Steroids	+	+	+	+

+: presence; −: absence.

**Table 2:** Total phenol, flavonoid, and tannin contents of plant extracts.

Plant extracts	TPC (mg·GAE/g)	TFC (mg·QE/g)	TTC (mg·TAE/g)
*T. mauritianum* (aerial parts)	110.23 ± 0.13^b^	7.32 ± 0.06^a^	10.90 ± 0.52^b^
*L. macrocarpa* (leaves)	57.89 ± 0.48^c^	9.16 ± 0.10^a^	4.08 ± 0.18^a^
*L. macrocarpa* (roots)	41.30 ± 0.39^d^	7.11 ± 0.10^b^	6.14 ± 0.13^a^
*G. bougheyanum* (aerial parts)	61.82 ± 0.14^e^	6.57 ± 0.14^a^	9.08 ± 0.62^b^

TPC: total phenolic content; TFC: total flavonoid content; TTC: total tannin content.

**Table 3:** Antiradical effect (IC_50_ in *µ*g/mL) of plant extracts scavenging DPPH-free radical.

IC_50_ (*µ*g/mL)				
*T. mauritianum* (aerial parts)	*L. macrocarpa* (leaves)	*L. macrocarpa* (roots)	*C. bougheyanum* (aerial parts)	Vitamin C
19.052 ± 1.11	59.48 ± 7.17	259.21 ± 83.06	23.70 ± 1.18	8.13 ± 1.78

**Table 4:** Hydroxyl-radical scavenging activity of plant extracts as a function of concentrations.

Plant extracts	Concentration (*µ*g/mL)				
	12.5	25	50	100	200
*T. mauritianum* (aerial parts)	3.73 ± 0.97^b^	4.99 ± 1.92^b^	9.07 ± 4.01^b^	20.98 ± 3.07^b^	66.001 ± 2.05^b^
*L. macrocarpa* (leaves)	1.69 ± 0.61^a^	2.01 ± 0.15^a^	4.83 ± 0.67^a^	15.90 ± 2.12^a^	22.73 ± 0.85^a^
*L. macrocarpa* (roots)	0.7 ± 0.95^a^	1.14 ± 1.05^a^	1.89 ± 0.99^a^	9.93 ± 1.75^a^	18.89 ± 2.67^a^
*G. bougheyanum* (aerial parts)	10.00 ± 0.83^c^	11.09 ± 0.03^d^	22.00 ± 0.82^d^	67.01 ± 2.13^d^	94.03 ± 1.26^e^
Vitamin C	2.07 ± 0.40^ab^	13.07 ± 0.70^e^	44.02 ± 0.35^e^	83.05 ± 4.00^e^	96.98 ± 0.90^e^

In each column, values with different letters are statistically different at *p* < 0.05 using Waller–Duncan test.

**Table 5:** Nitric oxide scavenging activity of plant extracts as a function of concentrations.

Plant extracts	Concentrations (*µ*g/mL)				
	12.5	25	50	100	200
*T. mauritianum* (aerial parts)	39.87 ± 10.10^a^	63.12 ± 6.76^ab^	70.07 ± 8.06^ab^	70.14 ± 18.31^a^	94.19 ± 3.54^a^
*L. macrocarpa* (leaves)	78.42 ± 14.67^b^	89.68 ± 4.06^b^	90.05 ± 2.34^c^	92.02 ± 1.04^c^	96.09 ± 1.02^b^
*L. macrocarpa* (roots)	62.71 ± 10.07^a^	69 ± 8.53^a^	74.09 ± 6.05^b^	83.74 ± 2.30^b^	86.93 ± 2.39^b^
*G. bougheyanum* (aerial parts)	50.61 ± 5.02^a^	66.01 ± 4.99^b^	79.98 ± 5.09^bc^	90.18 ± 5.04^b^	91.89 ± 1.69^b^
Vitamin C	92.03 ± 0.60^c^	94.25 ± 0.15^b^	96.06 ± 0.49^d^	97.02 ± 0.07^e^	99.09 ± 0.11^h^

In each column, values with different letters are statistically different at *p* < 0.05 using Waller–Duncan test.

**Table 6:** Antifungal activity (MIC and MFC in *μ*g/mL) of plant extracts and reference antifungal drugs against *Candida spp*.

Yeast species	*T. mauritianum* (aerial parts)		*L. macrocarpa* (leaves)		*L. macrocarpa* (roots)		*C. bougheyanum* (aerial parts)		Fluconazole		Ketoconazole	
	MIC	MFC	MIC	MFC	MIC	MFC	MIC	MFC	MIC	MFC	MIC	MFC
*C. dubliniensis* 5 : I52	256	1024	—	—	512	—	2048	—	256	—	16	128
*C. dubliniensis* 1 : I59	**64**	512	—	—	2048	—	512	—	—	—	256	—
*C. dubliniensis* 3 : I81	256	—	1024	—	1024	—	1024	—	256	—	256	—
*C. albicans* ATCC 10231	**64**	256	256	—	256	512	256	—	1	1	2	2
*C. albicans* 7Ca	128	1024	1024	—	512	2048	1024	—	256	—	128	256
*C. albicans* 11Ca	256	—	—	—	—	—	1024	—	—	—	16	64
*C. albicans* 18Ca	**64**	1024	512	—	256	2048	256	—	256	—	64	64
*C. albicans* Cppc BACT 017	256	—	2048	—	1024	—	1024	—	128	256	32	64
*C. glabrata* 11 : I81	128	—	2048	—	1024	—	1024		—	—	64	256
*C. glabrata* 10 : I39	128	—	1024	—	512	—	512	—	128		16	64
*C. tropicalis* Cpc BACT 018	**64**	2048	512	—	512	—	—	—	256	—	128	128

—: >2048 *µ*g/mL; MIC: minimum inhibitory concentration; MFC: minimum fungicidal concentration. Values in bold indicate the significant activity (MIC<100 *µ*g/mL).

**Table 7:** Interactions of ketoconazole with the plant extracts against yeast *Candida* isolates.

Yeast species	*T. mauritianum* (aerial parts)				*L. macrocarpa* (leaves)				*C. bougheyanum* (aerial parts)			
	FIC_A_	FIC_B_	∑FIC	Interpretation	FIC_A_	FIC_B_	∑FIC	Interpretation	FIC_A_	FIC_B_	∑FIC	Interpretation
*Candida dubliniensis* 5 : I52	0.125	0.125	0.25	Synergistic	0.015	<1	<2	Indifferent	0.015	0.25	0.3	Synergy
*Candida dubliniensis* 1 : I59	1	0.25	1.25	Indifferent	1	<1	<2	Indifferent	0.25	0.5	0.75	Additive
*Candida dubliniensis* 3 : I81	0.125	0.25	0.375	Synergistic	0.5	0.25	0.75	Additive	0.5	0.125	0.625	Additive
*Candida albicans* 7Ca	1	0.25	1.25	Indifferent	0.031	0.125	0.156	Synergistic	0.5	0.125	0.625	Additive
*Candida albicans* 11Ca	0.5	1	1.5	Indifferent	0.5	<1	1	Indifferent	0.25	1	1.25	Indifferent
*Candida albicans* 18Ca	0.25	0.125	0.375	Synergistic	0.5	0.25	0.75	Additive	1	0.25	1.25	Indifferent
*Candida albicans* Cppc BACT 017	0.25	0.25	0.25	Synergistic	0.25	0.125	0.375	Synergistic	0.5	0.5	1	Additive
*Candida glabrata* 11 : I81	0.5	1	1.5	Indifferent	0.25	0.125	0.375	Synergistic	0.25	0.125	0.375	Synergy
*Candida glabrata* 10 : I39	0.5	0.25	0.75	Additive	0.25	0.25	0.5	Synergistic	0.25	(0.25)	0.5	Synergy
*Candida tropicalis* Cpc BACT 018	1	0.25	1.25	Indifferent	0.25	0.125	0.375	Synergistic	0.25	<1	<2	Indifferent

FIC_A_: MIC of the ketoconazole tested in combination/MIC of ketoconazole tested alone; FIC_B_: MIC of the extract tested in combination/MIC of the extract tested alone; ∑FIC: FIC_A_ + FIC_E._

**Table 8:** Interactions of fluconazole with the plant extracts against yeast *Candida* isolates.

Yeast species	*T. mauritianum* (aerial parts)				*L. macrocarpa* (leaves)				*C. bougheyanum* (aerial parts)			
	FIC_A_	FIC_B_	∑FIC	Interpretation	FIC_A_	FIC_B_	∑FIC	Interpretation	FIC_A_	FIC_B_	∑FIC	Interpretation
*Candida dubliniensis* 5 : I52	0.25	0.25	0.5	Synergistic	0.125	<1	<2	Indifferent	0.062	0.062	0.124	Synergistic
*Candida dubliniensis* 1 : I59	<1	1	<2	Indifferent	<1	<1	<2	Indifferent	<1	1	<2	Indifferent
*Candida dubliniensis* 3 : I81	1	0.5	1.5	Indifferent	1	0.25	1.25	Indifferent	1	1	2	Indifferent
*Candida albicans* 7Ca	0.5	0.125	0.625	Additive	0.25	0.5	0.75	Additive	(0.5)	1	1.5	Additive
*Candida albicans* 11Ca	1	1	2	Indifferent	<1	<1	<2	Indifferent	<1	0.25	<2	Indifferent
*Candida albicans* 18Ca	0.5	0.25	0.75	Additive	0.125	0.25	0.375	Synergistic	1	0.25	1.25	Indifferent
*Candida albicans* Cppc BACT 017	0.25	0.25	0.5	Synergistic	0.25	0.25	0.5	Synergistic	1	1	2	Indifferent
*Candida glabrata* 11 : I81	<1	1	<2	Indifferent	<1	1	<2	Indifferent	<1	0.25	<2	Indifferent
*Candida glabrata* 10 : I39	1	1	2	Indifferent	(1)	1	2	Indifferent	0.25	0.25)	0.5	Synergistic
*Candida tropicalis* Cpc BACT 018	0.062	0.25	0.312	Synergistic	1	0.5	1.5	Additive	0.25	<1	<2	Indifferent

FIC_A_: MIC of the fluconazole tested in combination/MIC of the fluconazole tested alone; FIC_B_: MIC of the extract tested in combination/MIC of the extract tested alone; ∑FIC: FIC_A_ + FIC_E._

## Data Availability

The datasets generated and analysed during the current study are available from the corresponding author upon reasonable request.

## Abbreviations

DMSO: Dimethyl sulfoxide
DPPH: 2,2-Diphenyl-1-picrylhydrazyl
FRAP: Ferric reducing antioxidant power
GAE: Gallic acid equivalent
MFC: Minimal fungicidal concentration
MIC: Minimal inhibitory concentration
TCA: Trichloroacetic acid
TFC: Total flavonoid contents
TPC: Total polyphenolic contents.

## Supplementary Materials

Supplementary Materials Table S1: characteristics of *Candida spp*. used in the study [54] (supplementary material/table).
