# Supplementary material for: Phytochemical Analysis, Antifungal, and Antioxidant Properties of Two Herbs (Tristemma mauritianum and Crassocephalum bougheyanum) and One Tree (Lavigeria macrocarpa) Species
Source: Adv Pharmacol Pharm Sci. 2023 Jan 25;2023:2565857. doi: 10.1155/2023/2565857 (PMC9891821; doi:10.1155/2023/2565857)
Supplement: Supplementary Materials — Table S1: characteristics of Candida spp. used in the study [54] (supplementary material/table). [file 2565857.f1.pdf]

Phytochemical analysis, antifungal and antioxidant properties of two herb (*Tristemma mauritianum* and *Crassocephalum bougheyanum*) and one tree (*Lavigeria macrocarpa*) species

Irene Chinda Kengne[1], Aimé Gabriel Fankam[1], Elodie Konack Yamako[2], Jean-De-Dieu Tamokou[1]*

Supplementary material

List of Contents

Cover page 1

TABLE 1: Characteristics of *Candida* spp. used in the study 3

TABLE S1: Characteristics of *Candida spp*. used in the study [54]

Bacteria	Features
C. albicans ATCC 10231	Reference strain
C. dubliniensis 5:I52	Clinical isolate: Nys^r, Amph^r, Terb^r, Fluco^r, Keto^r
C. dubliniensis 1:I59	Clinical isolate: Fluco^r, Keto^r
C. dubliniensis 3:I81	Clinical isolate: Nys^r, Fluco^r, Keto^r
C. albicans 7Ca	Clinical isolate: Nys^r, Amph^r, Fluco^r, Keto^r
C. albicans 11Ca	Clinical isolate: Nys^r, Terb^r, Fluco^r, Keto^r
C. albicans 18Ca	Clinical isolate: Nys^r, Fluco^r, Keto^r
C. albicans Cppc BACT 017	Clinical isolate : Nys^r, Amph^r, Fluco^r, Keto^r
C. glabrata 11:I81	Clinical isolate : Fluco^r, Keto^r
C. glabrata 10:I39	Clinical isolate : Nys^r, Fluco^r, Keto^r
C. tropicalis Cpc BACT 018	Clinical isolate : Amph^r, Terb^r, Fluco^r, Keto^r

Nys: nystatin, Amph: amphotericine B, Terb: terbinafine, Fluco: fluconazole, Keto: ketonazole. R: resistant
